# TGFβ1 and SMAD3 Expression Are Associated With Survival After the Immune Checkpoint Inhibitor Therapy for Small Cell Lung Cancer

**DOI:** 10.1002/cnr2.70394

**Published:** 2025-11-04

**Authors:** Zenta Seto, Minehiko Inomata, Akira Noguchi, Tomonobu Kado, Nozomu Murayama, Naoki Takata, Kotaro Tokui, Seisuke Okazawa, Shingo Imanishi, Tosihiro Miwa, Ryuji Hayashi, Kenichi Hirabayashi, Kazuyuki Tobe

**Affiliations:** ^1^ First Department of Internal Medicine Toyama University Hospital Toyama Japan; ^2^ Department of Diagnostic Pathology, Faculty of Medicine, Academic Assembly University of Toyama Toyama Japan; ^3^ Department of Clinical Oncology Toyama University Hospital Toyama Japan

**Keywords:** immune checkpoint inhibitor, SMAD3, small cell lung cancer, TGFβ

## Abstract

**Background:**

Tumor immunity is involved in the progression of malignant tumors. However, there are few reports on the relationship between the immunological environment and the efficacy of immune checkpoint inhibitors in small cell lung cancer (SCLC). We analyzed the relationship between tumor‐infiltrating immune cells and protein expression and survival in patients with SCLC treated with combined therapy with immune checkpoint inhibitors plus chemotherapy.

**Methods:**

Patients with SCLC who received combined therapy with immune checkpoint inhibitors plus chemotherapy between 2019 and 2023 were enrolled. Immune cell infiltration levels, including CD4, CD8, FOXP3, CD163‐positive cells and expression levels of TGFβ1 and SMAD3 proteins in tumor tissue were evaluated by immunohistochemistry. Progression‐free survival (PFS) and overall survival (OS) were evaluated as endpoints.

**Results:**

Data from 22 patients were analyzed. The high CD4‐positive T lymphocyte (*p* = 0.008, log‐rank test) and the high CD8‐positive T lymphocyte infiltration group (*p* = 0.031, log‐rank test) showed statistically significantly longer OS than the low infiltration group. On the other hand, the low TGFβ1 expression group showed significantly longer PFS (*p* = 0.026, log‐rank test) and the low SMAD3 expression group showed significantly longer OS (*p* = 0.042, log‐rank test) than the high expression group.

**Conclusion:**

In conclusion, it is suggested that infiltration of T lymphocytes and expression of TGFβ1 and SMAD3 may be related to the efficacy of the combined therapy with immune checkpoint inhibitors plus chemotherapy in patients with SCLC.

## 1 Introduction

Small cell lung cancer (SCLC) accounts for 13% of all lung cancers, and it is known for its rapid progression. Advanced SCLC responds well to frontline cytotoxic chemotherapy, but many patients have progressive disease and have a median survival of less than 1 year after initiation of cytotoxic chemotherapy [1].

Recently, immune checkpoint inhibitors have been developed and used for many types of cancer. Although immune checkpoint inhibitor monotherapy has not shown the efficacy over cytotoxic chemotherapy in SCLC [2], combined therapy with immune checkpoint inhibitors plus chemotherapy showed an overall survival (OS) rate superior to that of conventional cytotoxic agents for advanced SCLC [3, 4]. However, survival rate gains were small, and early deaths are still common. Therefore, there is a need to improve the efficacy of immune checkpoint inhibitors in advanced SCLC.

Tumor immunity is involved in the progression of malignant tumors. In non‐small cell lung cancer (NSCLC), tumor‐infiltrating T lymphocytes have been shown to be associated with extended survival in patients treated with immune checkpoint inhibitors [5, 6, 7, 8]. On the other hand, tumor infiltration of monocytes is also observed to a high degree in NSCLC [9]. Macrophages in particular have been reported to be one of the immune cells that promote tumor progression, and they are classified into M1 and M2 from a functional perspective. Macrophages in tumor tissues are polarized into M2 macrophages and promote the survival and proliferation of cancer cells through the production of vascular endothelial growth factor (VEGF), epidermal growth factor, and transforming growth factor (TGF)β1 [10]. Consistent with this, clinical studies have reported a relationship between tumor‐infiltrating macrophages in epidermal growth factor receptor mutant NSCLC and shortened progression‐free survival (PFS) after initiation of immune checkpoint inhibitor treatment [11], and a relationship between tumor‐infiltrating M2 macrophages in NSCLC and shortened PFS after initiation of immune checkpoint inhibitor treatment [6]. TGFβ and its downstream effector SMAD3 play a dual role in tumor development [12]. In the early stages of tumor progression, this signaling pathway functions as a tumor suppressor by inducing apoptosis. In contrast, during the later stages, it promotes tumor progression through multiple mechanisms, including epithelial–mesenchymal transition [12], induction of M2 macrophage polarization, enhancement of the immunosuppressive properties of cancer‐associated fibroblasts [13], and polarization of tumor‐associated neutrophils toward a pro‐tumor phenotype [14].

However, there are few reports on the relationship between the immunological environment and the therapeutic effect of immune checkpoint inhibitors in SCLC [15, 16]. Analysis of the tumor microenvironment may contribute to improving the therapeutic effect of immune checkpoint inhibitors or developing new treatments. We analyzed the relationship between tumor‐infiltrating immune cells (CD4, CD8, FOXP3, CD163‐positive cells) and proteins (TGFβ1 and its downstream signal, SMAD3) expression and survival after the start of combined therapy with immune checkpoint inhibitors plus chemotherapy in SCLC.

## 2 Methods

### 2.1 Study Design and Patient Selection

This study was conducted retrospectively. Selection criteria were as follows: (1) patients who were diagnosed cytologically or histologically as having SCLC or combined small‐cell carcinoma; (2) patients who were treated with combined therapy with immune checkpoint inhibitors plus chemotherapy between August 2018 and April 2023, including patients who were previously treated with postoperative adjuvant chemotherapy or chemoradiotherapy. Exclusion criteria were as follows: (1) patients in whom the pathological specimens were not available; (2) patients who were diagnosed by biopsy from lymph node, bone marrow, brain, or pleural effusion cell block.

The present study was conducted in accordance with the Declaration of Helsinki and the Ethical Guidelines for Medical and Biological Research Involving Human Subjects (Ministry of Health, Labor and Welfare, Japan). The study plan was approved by the Ethics Committee, University of Toyama (approval number: R2023064). Since the present study used existing information and biopsy samples without any intervention or invasion, the need to obtain individual informed consent was waived, and research information was made available to research participants.

### 2.2 Treatment and Clinical Information

Therapeutic regimen and treatment schedule were decided by physicians based on clinical judgment. Clinical information including patient demographics, histology, therapeutic regimen, date of disease progression, and date of last visit was collected from medical records.

### 2.3 Immunohistochemistry

The following antibody was used for immunohistochemistry: anti‐CD4 mouse monoclonal antibody (clone: 4B12, NCL‐L‐CD4‐368, Leica Biosystems Inc., Deer Park, US), anti‐CD8 mouse monoclonal antibody (clone: 4B11, NCL‐L‐CD8‐4B11, Leica Biosystems Inc., Deer Park, US), anti‐FOXP3 mouse antibody (clone: 236A/E7, ab20034, Abcam Inc., Cambridge, UK), anti‐CD163 mouse monoclonal antibody (clone: 10D6, NCL‐L‐CD163, Leica Biosystems Inc., Deer Park, US), anti‐TGFβ1 rabbit monoclonal antibody (clone: EPR21143, ab179695, Abcam Inc., Cambridge, UK), anti‐SMAD3 rabbit monoclonal antibody (clone: EP823Y, ab52903, Abcam Inc., Cambridge, UK). Deparaffinization, antigen retrieval treatment, endogenous peroxidase blocking were performed using Leica BOND‐MAX (Leica Biosystems Inc., Deer Park, US) according to the manufacturer's instructions.

We used tissue biopsies taken before the treatment. Immune cell infiltration level and protein expression level were evaluated by the pathologist and semi‐quantitatively scored according to immunohistochemistry findings as a four‐step grade, based on the agreement with two pulmonary physicians. The antigen with a more widely distributed reactivity was given the value 3 (strong). The antigen showing a more limited reactivity was given the value 1 (weak) or 2 (moderate), depending on the difference in the reactivities between the antigens. Subsequently, considering the number of cases, two pulmonary physicians classified the patients into two groups based on immunohistochemical scoring. The cutoff values were chosen to minimize the imbalance in patient numbers between groups. When assessing tissue sections, the observers were blinded to each other's scores, clinical variables and patient's outcome.

### 2.4 Statistical Analysis

The endpoints of the present study were PFS and OS from the initiation of combined therapy with immune checkpoint inhibitors plus chemotherapy. PFS was calculated from the date of treatment initiation until either disease progression or death was observed. Disease progression was defined as progressive disease according to the Response Evaluation Criteria in Solid Tumors version 1.1 or progression based on clinical judgment. PFS was censored at the last event‐free visit. OS was calculated from the date of treatment initiation until the date of death, and censored at the last event‐free visit.

Kaplan–Meier curve was drawn and survival time was compared using the log‐rank test. Demographic characteristics of patients were compared using Fisher's exact test. A *p* value less than 0.05 was considered significant. All statistical analyses were performed with JMP version 17.0.0 (SAS, Cary, US).

## 3 Results

Between 2019 and 2023, 35 patients with SCLC received combined therapy with immune checkpoint inhibitors plus chemotherapy. Two patients were excluded because pathological specimens were unavailable, and 11 patients were excluded because their diagnosis was based on lymph node biopsy, bone marrow biopsy, or pleural effusion cell block. Finally, 22 patients were analyzed (Table 1). Twenty‐one and one patient were diagnosed with SCLC by biopsy from the lung, liver, and chest wall, respectively. Twenty‐one of 22 patients had a PS 0–1. Ten patients were treated with atezolizumab plus platinum‐based chemotherapy and etoposide, and 12 patients were treated with durvalumab plus platinum‐based chemotherapy and etoposide.

**TABLE 1:** Patient characteristics.

		*n*	%
Sex	Male	18	81.8
	Female	4	18.2
Age (years)	< 70	7	31.8
	≥ 70	15	68.2
PS	0	5	22.7
	1	16	72.7
	3	1	4.5
Biopsy site	Lung	20	90.9
	Liver	1	4.5
	Chest wall	1	4.5
Treatment	CBDCA+VP‐16 + atezolizumab	10	45.5
	CBDCA+VP‐16 + durvalumab	10	45.5
	CDDP+VP‐16 + durvalumab	2	9.1
Disease stage	Stage IVA	3	13.6
	Stage IVB	12	54.5
	Recurrence	7	31.8
Treatment history^a^	Radical surgery	4	18.2
	Chemoradiation	4	18.2
	None	15	68.2
Brain metastasis	Yes	3	13.6
	No	19	86.4

Abbreviations: CBDCA, carboplatin; CDDP, cisplatin; PS, performance status; VP‐16, etoposide.

^a^ One patient received both of surgery and chemoradiation.

**FIGURE 1:**
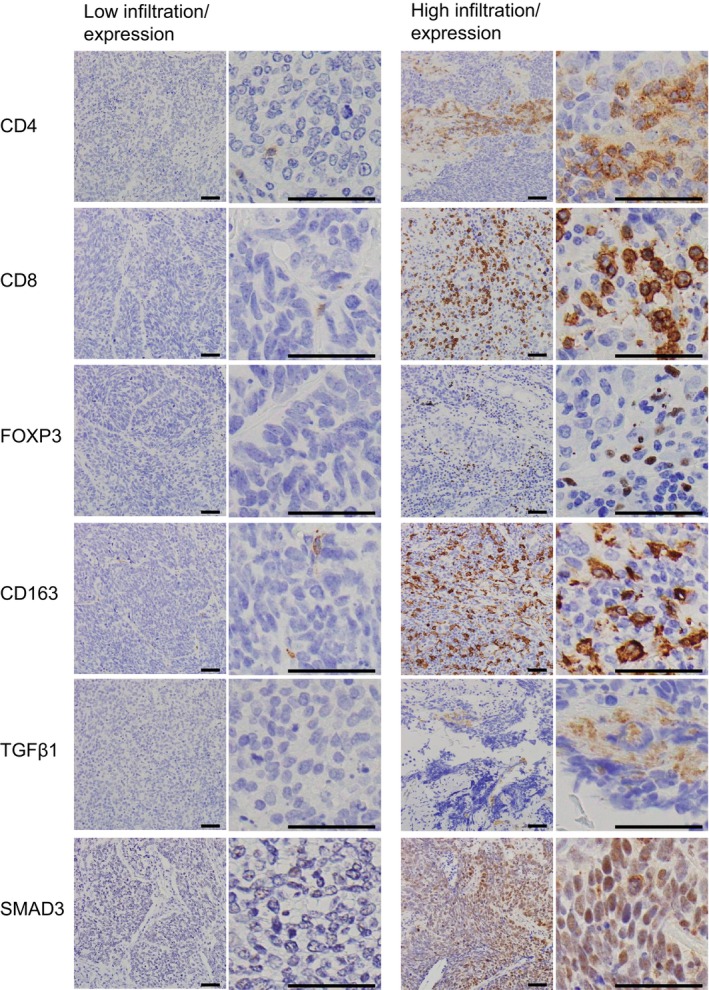
Immunohistochemical findings of the representative sample. Black bar indicates 50 μm. Immune cell and protein expression were classified into low‐ or high‐infiltration (expression) groups according to the semi‐quantitative scoring. TGFβ1 was detected in the stroma and SMAD3 was detected in mainly tumor cells.

Figure 1 shows the immunohistochemical findings of representative examples. CD4‐ and CD8‐positive T lymphocytes infiltrated the tumor tissue at various levels, and specimens with negative or value 1 (weak) staining were classified as the low‐infiltration group, while those with value 2 (moderate) or 3 (strong) staining were classified as the high‐infiltration group. CD163‐positive macrophages were observed in all specimens, and similarly, specimens with value 1 (weak) staining were classified as the low‐infiltration group, while those with value 2 (moderate) or 3 (strong) were assigned to the high‐infiltration group. The proportion of cases with high CD163‐positive cell infiltration was statistically significantly higher in the group with high CD8‐positive T lymphocyte infiltration (100% vs. 23.1%, *p* < 0.001, Fisher's exact test). For FOXP3, TGFβ1, and SMAD3, specimens with negative staining were categorized as the low‐infiltration/expression group, whereas those with value 1 (weak) or stronger staining were classified as the high‐infiltration/expression group. TGFβ1 was positive in the stroma, and SMAD3 was positive in tumor cells.

**FIGURE 2:**
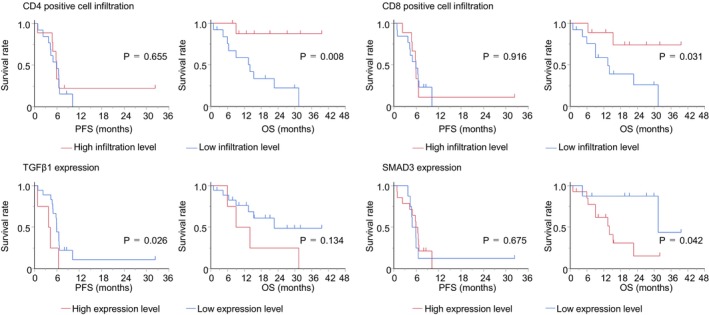
The comparison of progression‐free survival and overall survival in the patients subdivided according to the infiltration or expression level of immune cells and protein (log‐rank test). High CD4/CD8 T‐cell infiltration was significantly associated with improved overall survival. Patients with low TGFβ1 expression had significantly longer progression‐free survival and showed a trend toward improved overall survival. Similarly, low SMAD3 expression was significantly associated with longer overall survival.

Table 2 shows the relationship between immune cell infiltration and protein expression level to PFS and OS after the start of combination therapy with immune checkpoint inhibitors plus chemotherapy. The high CD4‐positive T lymphocyte infiltration group (median: not reached, NR vs. 14.0 months, *p* = 0.008, log‐rank test) and the high CD8‐positive T lymphocyte infiltration group (median: NR vs. 13.4 months, *p* = 0.031, log‐rank test) show statistically significantly longer OS than the low infiltration group. The low TGFβ1 expression group shows significantly longer PFS than the high TGFβ1 expression group (median 5.9 vs. 4.0 months, *p* = 0.026, log‐rank test). Although the difference was not statistically significant, OS tended to be longer in the low TGFβ1 expression group than in the high expression group. The low SMAD3 expression group shows significantly longer OS than the high SMAD3 expression group (median 31.4 vs. 14.0 months, *p* = 0.042, log‐rank test) (Figure 2).

**TABLE 2:** The association of immune cell infiltration level or cytokine expression level with progression‐free survival and overall survival (log‐rank test).

		Progression‐free survival			Overall survival		
		Median	95% CI	*p*	Median	95% CI	*p*
CD4	High	5.9	0.9–NE	0.655	NR	9.0–NE	0.008
	Low	5.9	3.8–6.5		14.0	4.4–22.6	
CD8	High	5.9	2.3–6.6	0.916	NR	6.3–NE	0.031
	Low	5.9	3.8–6.7		13.4	4.4–31.4	
FOXP3	High	5.9	2.3–6.6	0.833	31.4	9.0–NE	0.135
	Low	5.8	4.3–6.7		13.4	4.4–NE	
CD163	High	5.9	2.3–6.6	0.876	NR	9.0–NE	0.063
	Low	5.7	0.9–6.7		13.4	0.9–31.4	
TGFβ1	High	4.0	0.9–6.4	0.026	11.5	5.9–31.4	0.134
	Low	5.9	4.9–6.6		22.6	9.0–NE	
SMAD3	High	6.2	2.3–6.7	0.675	14.0	6.3–22.6	0.042
	Low	5.4	3.8–6.5		31.4	4.4–NE	

Abbreviations: CI, confidential interval; NE, not estimated; NR, not reached; TGF, transforming growth factor.

Table 3 shows the results of comparing the clinical and immunological characteristics between the low and high TGFβ1 expression groups. There were no significant differences in patient characteristics or immune profiles between the groups with higher and lower TGFβ1 expression. Similarly, we found no significant differences in patient characteristics or immune profiles with regard to SMAD3 expression (Table S1).

**TABLE 3:** The association between TGFβ1 expression level and clinical or immunologic parameters.

		TGFβ1 expression level				*p*
		Low		High		
Sex	Male	16	88.9%	2	50.0%	0.136
	Female	2	11.1%	2	50.0%	
Age (years)	< 70	6	33.3%	1	25.0%	1.000
	≥ 70	12	66.7%	3	75.0%	
PS	0	5	27.8%	0	0.0%	0.617
	1	12	66.7%	4	100.0%	
	3	1	5.6%	0	0.0%	
Biopsy site	Lung	16	88.9%	4	100.0%	1.000
	Liver	1	5.6%	0	0.0%	
	Chest wall	1	5.6%	0	0.0%	
Treatment	CBDCA+VP‐16 + atezolizumab	8	44.4%	2	50.0%	0.395
	CBDCA+VP‐16 + durvalumab	9	50.0%	1	25.0%	
	CDDP+VP‐16 + durvalumab	1	5.6%	1	25.0%	
CD4	High	8	44.4%	1	25.0%	0.616
	Low	10	55.6%	3	75.0%	
CD8	High	9	50.0%	0	0.0%	0.115
	Low	9	50.0%	4	100.0%	
FOXP3	High	8	44.4%	3	75.0%	0.587
	Low	10	55.6%	1	25.0%	
CD163	High	11	61.1%	1	25.0%	0.293
	Low	7	38.9%	3	75.0%	
SMAD3	High	7	38.9%	1	25.0%	1.000
	Low	11	61.1%	3	75.0%	

Abbreviations: CBDCA, carboplatin; CDDP, cisplatin; PS, performance status; TGF, transforming growth factor; VP‐16, etoposide.

## 4 Discussion

The present study showed that infiltration/expression levels of CD4 and CD8‐positive T lymphocytes and TGFβ1 and SMAD3 were associated with survival from the initiation of the combined therapy with chemotherapy plus immune checkpoint inhibitors in patients with SCLC. Many previous studies have reported the role of tumor‐infiltrating T lymphocytes in tumor immunity, and the present study confirmed their influence on the clinical course of SCLC. As for TGFβ1 and SMAD3, several preclinical studies and clinical trials have been conducted to develop novel treatments for solid tumors [17]. It is expected that TGFβ1 and SMAD3 may be the new treatment targets in SCLC from the present study.

In SCLC, 80% of cases exhibit high expression of achaete‐scute homolog 1 (ASCL1) and neurogenic differentiation factor 1 (NEUROD1), which are genes involved in transcriptional regulation. These genes are associated with the neuroendocrine characteristics of the cancer and the reduction of T lymphocyte infiltration within tumor tissue [18]. Conversely, some cases show increased expression of POU class 2 homeobox (POU2F3) and human leukocyte antigen (HLA), and it has been reported that the expression of these genes correlates with the expression of immune‐related genes and enhanced T cell infiltration [18].

Tumor‐infiltrating CD8‐positive T lymphocytes contribute to tumor cell exclusion. The previous meta‐analysis including several malignancies showed the positive effect of tumor‐infiltrating T lymphocytes on the OS [19]. In patients with SCLC, they have been reported to be associated with longer OS after surgery [20] and PFS after initiation of immune checkpoint inhibitor therapy [15, 16]. The present study showed the statistically significant association between the CD8‐positive T lymphocytes infiltration level and OS from the treatment initiation. Based on these findings, it is suggested that tumor‐infiltrating CD8‐positive T lymphocytes are associated with both the natural course of patients with SCLC and the efficacy of immune checkpoint inhibitor therapy.

CD4‐positive T lymphocytes are also considered to play an important role in tumor immunity. Although the interaction between CD4‐positive T lymphocytes and CD8‐positive T lymphocytes in tumor immunity is only partially understood, it is possible that dendritic cells function as a platform that supports the interaction between CD4 and CD8 T lymphocytes, and that CD4‐positive T lymphocytes may have an important function in the expression of the anti‐tumor effects of CD8‐positive T lymphocytes [21]. In the clinical settings, it has been reported that tumor‐infiltrating CD4‐positive T lymphocytes [8], peripheral CD62 low CD4‐positive T lymphocytes [22], and peripheral CD4‐positive memory T lymphocytes [23] are associated with the efficacy of immune checkpoint inhibitors in patients with NSCLC.

CD163 is one of the M2 macrophage marker [24]. It has been reported that tumor infiltration of CD163‐positive macrophages is associated with shortened PFS after the treatment initiation of immune checkpoint inhibitors in patients with NSCLC [6], and shortened OS of patients who were diagnosed as having SCLC between 2017 and 2020 [25]. However, the present study failed to show the association between tumor‐infiltrating CD163‐positive macrophages and survival in patients with SCLC. It may be hypothesized that M2 macrophages have a little effect on the immune checkpoint inhibitor efficacy in SCLC, but there was an overlap of infiltration of CD8‐positive lymphocytes and CD163‐positive macrophages, which might affect the analysis in our study. Li et al. reported that joint parameters such as CD8+ high/CD68 + CD163+ low were more predictive for PFS after the treatment initiation with immune checkpoint inhibitors [6]. However, due to the small sample size, we could not perform an analysis using joint parameters or subset analysis.

TGFβ works tumor‐suppressive at the early phase of tumor progression, and in the late phase, it promotes tumor progression via metastasis, angiogenesis, or epithelial‐mesenchymal transition [12]. Clinical study of NSCLC showed that increased plasma TGFβ expression was associated with resistance against immune checkpoint inhibitors [26]. Similarly, it has been shown that increased expression of TGFβ1 and SMAD3, the downstream of TGFβ1, was associated with shorter PFS or OS in patients with SCLC who were treated with immune checkpoint inhibitors in the present study. However, opposite findings were also reported from the studies of SCLC [27, 28]. Zhang J et al. analyzed data from 90 cases of SCLC that underwent curative resection and reported that the expression of TGFβ1 in cancer‐associated fibroblasts, assessed by immunostaining, was associated with longer postoperative OS [27]. Given that this study focused on curatively resected SCLC, the results may have been influenced by the tumor progression inhibitory effect of TGFβ in the early stages of the disease. Lin A et al. reported that elevated TGFβ expression, determined via RNA sequencing, correlated with longer OS following the initiation of cytotoxic anticancer drugs [28]. This study highlighted a reduction in M2 macrophages within tumor tissue, upregulation of the apoptosis pathway, and downregulation of the DNA repair pathway in the TGFβ‐enhanced group [28], which may have impacted the results of this study. The influence of TGFβ on the clinical course of SCLC is considered not fully elucidated.

Although macrophage is one of the sources of TGFβ production, the present study failed to show the association between the expression of TGFβ1 and the infiltration level of CD163‐positive macrophage. In addition to macrophages, tumor cells are also known to be a source of TGFβ [10]. However, because there may be the issue of sensitivity of immunohistochemistry, it is difficult to discuss the production source of TGFβ1 based on the findings of the present study.

The present study has several limitations. First, study participants were only 22; it is unclear whether this population is representative of general SCLC patients. Furthermore, although it cannot be excluded that accidental imbalance of patient characteristics may influence the analysis, it was difficult to perform the multivariate analysis due to the small sample size. Third, although the immunohistochemical results in this study were classified using a semi‐quantitative method, concerns remain regarding the reproducibility and objectivity. Several scoring systems have been proposed for immunohistochemical analysis, including the Immunoreactive Score and the H‐score, among others [29]. Further studies are warranted to clarify the impact of TGFβ1 and SMAD3 expression on the clinical course of patients with SCLC. Finally, because the infiltration of CD8‐positive T cells and CD163‐positive macrophages overlapped, it was difficult to analyze the association between these immune cells and survival separately.

In summary, the influence of the infiltration and expression of CD4/CD8‐positive T lymphocytes, TGFβ1, and SMAD3 on the effectiveness of the combined therapy with immune checkpoint inhibitor plus chemotherapy is suggested. Given the association of TGFβ1 and SMAD3 with PFS or OS, the novel treatment strategy targeting these molecules is expected for SCLC.

## Author Contributions

Minehiko Inomata and Tomonobu Kado designed the study. Akira Noguchi performed immunohistochemistry. Zenta Seto, Minehiko Inomata, Tomonobu Kado, Kenichi Hirabayashi, and Kazuyuki Tobe contributed to the interpretation of immunohistochemistry findings. Naoki Takata, Nozomu Murayama, Kotaro Tokui, Seisuke Okazawa, Shingo Imanishi, Toshiro Miwa, and Ryuji Hayashi contributed to the data collection. Zenta Seto and Minehiko Inomata prepared the original draft.

## Ethics Statement

This study was performed in line with the principles of the Declaration of Helsinki. Approval was granted by the Ethics Committee of University of Toyama (Ethics Committee, University of Toyama, approval number: R2023064).

## Consent

The need to obtain individual informed consent was waived, and research information was made available to research participants.

## Conflicts of Interest

The authors declare no conflicts of interest.

## Supporting information


**Table S1:** The association between SMAD3 expression level and clinical or immunologic parameters.

## Data Availability

The datasets analyzed during the current study are available from the corresponding author on reasonable request.
