# Supplementary material for: TGFβ1 and SMAD3 Expression Are Associated With Survival After the Immune Checkpoint Inhibitor Therapy for Small Cell Lung Cancer
Source: Cancer Rep (Hoboken). 2025 Nov 4;8(11):e70394. doi: 10.1002/cnr2.70394 (PMC12585284; doi:10.1002/cnr2.70394)
Supplement: Supplementary file 1 — Table S1: The association between SMAD3 expression level and clinical or immunologic parameters. [file CNR2-8-e70394-s001.docx]

Table S1. The association between SMAD3 expression level and clinical or immunologic parameters.

|  |  | SMAD3 expression level | | | |  |
| --- | --- | --- | --- | --- | --- | --- |
|  |  | Low | | High | | P |
| Sex | Male | 6 | 75.0% | 12 | 85.7% | 0.602 |
|  | Female | 2 | 25.0% | 2 | 14.3% |  |
| Age (years) | < 70 | 1 | 12.5% | 6 | 42.9% | 0.193 |
|  | ≥ 70 | 7 | 87.5% | 8 | 57.1% |  |
| PS | 0 | 2 | 25.0% | 3 | 21.4% | 0.571 |
|  | 1 | 5 | 62.5% | 11 | 78.6% |  |
|  | 3 | 1 | 12.5% | 0 | 0.0% |  |
| Biopsy site | Lung | 8 | 100.0% | 12 | 85.7% | 1.000 |
|  | Liver | 0 | 0.0% | 1 | 7.1% |  |
|  | Chest wall | 0 | 0.0% | 1 | 7.1% |  |
| Treatment | CBDCA+VP-16+atezolizumab | 4 | 50.0% | 6 | 42.9% | 0.685 |
|  | CBDCA+VP-16+durvalumab | 4 | 50.0% | 6 | 42.9% |  |
|  | CDDP+VP-16+durvalumab | 0 | 0.0% | 2 | 14.3% |  |
| CD4 | High | 4 | 50.0% | 5 | 35.7% | 0.662 |
|  | low | 4 | 50.0% | 9 | 64.3% |  |
| CD8 | High | 5 | 62.5% | 4 | 28.6% | 0.187 |
|  | low | 3 | 37.5% | 10 | 71.4% |  |
| FOXP3 | High | 7 | 50.0% | 4 | 50.0% | 1.000 |
|  | low | 7 | 50.0% | 4 | 50.0% |  |
| CD163 | High | 5 | 62.5% | 7 | 50.0% | 0.675 |
|  | low | 3 | 37.5% | 7 | 50.0% |  |
| TGFβ1 | High | 1 | 12.5% | 3 | 21.4% | 1.000 |
|  | low | 7 | 87.5% | 11 | 78.6% |  |

CBDCA, carboplatin; CDDP, cisplatin; PS, performance status; TGF, transforming growth factor; VP-16, etoposide.
